# Complete Genome Sequence of Carbapenemase-Producing *Klebsiella pneumoniae* Myophage Matisse

**DOI:** 10.1128/genomeA.01136-15

**Published:** 2015-10-01

**Authors:** Vincent E. Provasek, Lauren E. Lessor, Jesse L. Cahill, Eric S. Rasche, Gabriel F. Kuty Everett

**Affiliations:** Center for Phage Technology, Texas A&M University, College Station, Texas, USA

## Abstract

*Klebsiella pneumoniae* is a leading cause of nosocomial infections in the United States. Due to the emergence of multidrug-resistant strains, phages targeting *K. pneumoniae* may be a useful alternative against this pathogen. Here, we announce the complete genome of *K. pneumoniae* pseudo-T-even myophage Matisse and describe its features.

## GENOME ANNOUNCEMENT

*Klebsiella pneumoniae* is a Gram-negative opportunistic pathogen in the family *Enterobacteriaceae*. An important interest in *K. pneumoniae* lies in its cause of nosocomial infections, wherein it accounts for approximately 8% of all hospital-acquired infections in the United States (1). The emergence of multidrug-resistant *K. pneumoniae* carbapenemase-producing (KPC) strains of *K. pneumoniae* carries with it the problem of limited antibiotic choice (2, 3). As an alternative, the use of bacteriophage biocontrol to treat resistant infections has been used in the past and continues to gain momentum (4). Here, we describe pseudo-T-even bacteriophage Matisse isolated against KPC-producing *K. pneumoniae* strain A1.

Bacteriophage Matisse was isolated from a sewage sample collected at College Station, TX. Phage DNA was sequenced in an Illumina MiSeq 250-bp paired-end run with a 550-bp insert library at the Genomic Sequencing and Analysis Facility at the University of Texas (Austin, TX). Quality controlled trimmed reads were assembled to a single contig of circular assembly at 34.9-fold coverage using SPAdes version 3.5.0 (5). The contig was confirmed to be complete by PCR using primers that face the upstream and downstream ends of the contig. Products from the PCR amplification of the junctions of concatemeric molecules were sequenced by Sanger sequencing (Eton Bioscience, San Diego, CA). Genes were predicted using GeneMarkS (6) and corrected using software tools available on the Center for Phage Technology (CPT) Galaxy instance (https://cpt.tamu.edu/galaxy-public/). The morphology of Matisse was determined using transmission electron microscopy performed at the Texas A&M University Microscopy and Imaging Center.

Matisse is a pseudo-T-even myophage with a 176,081-bp genome, a coding density of 95.7%, and G+C content of 41.8%. Genomic analysis and annotation of Matisse showed 280 predicted coding sequences, of which 110 have a predicted function by BLASTp, InterProScan, and CD-search analyses (7–9). Like other pseudo-T-even phages, Matisse is opened to the *rIIb* gene due to overlap of the start and stop codons of *rIIb* and *rIIa*, respectively (10). Matisse shows 64.5% and 95.3% nucleotide sequence identity across the genome to pseudo-T-even bacteriophages RB43 (accession no. NC_007023) and KP15 (accession no. NC_014036), respectively, as determined by Emboss Stretcher (11). It belongs to the Lytic2 T4 subcluster-I recently described by Grose and Casjens (12). Matisse encodes one tRNA (Met) and five homing endonucleases, two of which contain an AP2 DNA-binding domain (13).

A bifunctional Nudix hydrolase/NMN adenylyltransferase was identified that is distinct from the monofunctional Nudix hydrolase, NudE, previously described in T4 (14). This bifunctional protein is common among pseudo-T-even phages and appears to be an ortholog of a pyridine salvage pathway protein previously described in T4-like *Vibrio* phage KVP40 (14–16). Interestingly, an open reading frame encoding a homolog of the T4-like NudE protein was also predicted in the Matisse genome. It is unclear, however, if these observations reflect functional redundancy.

### Nucleotide sequence accession number.

The genome sequence of phage Matisse was contributed as accession no. KT001918 to GenBank.
